# Cortical tau burden and behavioural dysfunctions in mice exposed to monosodium glutamate in early life

**DOI:** 10.1371/journal.pone.0220720

**Published:** 2019-08-14

**Authors:** Passainte S. Hassaan, Abeer E. Dief, Teshreen M. Zeitoun, Azza M. Baraka, Robert M. J. Deacon, Amany Elshorbagy

**Affiliations:** 1 Department of Medical Physiology, Faculty of Medicine, University of Alexandria, Alexandria, Egypt; 2 Department of Medical Histology and Cell Biology, Faculty of Medicine, University of Alexandria, Alexandria, Egypt; 3 Department of Clinical Pharmacology, Faculty of Medicine, University of Alexandria, Alexandria, Egypt; 4 Basic Sciences Division, Faculty of Medicine, University of Alexandria, Alexandria, Egypt; McGill University, CANADA

## Abstract

Although monosodium glutamate (MSG)-induced neurotoxicity has been recognized for decades, the potential similarities of the MSG model to Alzheimer’s disease (AD)-type neuropathology have only recently been investigated. MSG-treated mice were examined behaviourally and histologically in relation to some features of AD. Four-week old mice received 5 subcutaneous MSG (2 g/kg) injections on alternate days, or saline. At age 10–12 weeks, they were given a battery of behavioural tests for species-typical behaviours and working memory. The mice were killed at 12 weeks and the brains excised. Accumulation of hyperphosphorylated tau protein was assessed in cortical and hippocampal neurons by immunohistochemistry, and in cerebral cortical homogenates. A 78% increase in cortical concentrations of phosphorylated tau protein was observed in the MSG mice. Intracellular hyperphosphorylated tau immunostaining was observed diffusely in the cortex and hippocampus, together with cortical atrophic neurons, extensive vacuolation and dysmorphic neuropil suggestive of spongiform neurodegeneration. Nest-building was significantly impaired, and spontaneous T-maze alternation was reduced, suggesting defective short-term working memory. Subcutaneous MSG treatment also induced a 56% reduction in exploratory head dips in a holeboard (P = 0.009), and a non-significant tendency for decreased burrowing behaviour (P = 0.085). These effects occurred in the absence of MSG-induced obesity or gross locomotor deficits. The findings point to subcutaneous MSG administration in early life as a cause of tau pathology and compromised species-typical behaviour in rodents. Determining whether MSG can be useful in modelling AD requires further studies of longer duration and full behavioural characterization.

## Introduction

Alzheimer’s disease (AD) is the most common cause of dementia, a devastating condition affecting 50 million people worldwide, and causing significant disability and dependency [1]. Aggregation of intraneuronal hyperphosphorylated forms of the microtubule-associated tau protein in the form of neurofibrillary tangles is a hallmark of the disease, and parallels cognitive dysfunction in AD patients [2,3]. To date, there is no treatment for AD, despite some successes in preventive strategies [4]. Numerous transgenic and pharmacologic animal models of AD have been developed to test potential treatments, but a perfect model capturing all AD features remains elusive [5].

Although monosodium glutamate (MSG)-induced neurotoxicity has been recognized for decades [6], the potential similarities of the MSG model to AD-type neuropathology have only recently been investigated. Increased β-amyloid, coupled with memory and behavioural deficits, was seen in the hippocampus of rats treated with MSG [7]. Subsequently, hippocampal hyperphosphorylated tau was also detected in a mouse model of MSG-induced obesity [8]. The tau hyperphosphorylation was prevented by treatment with liraglutide, an anorexigenic and anti-diabetic drug [8]. Therefore, MSG-induced neurotoxicity may be a useful model for studying AD-like neuropathology and potential therapies. MSG neurotoxicity can be induced by subcutaneous or oral dosing [9], which is technically less demanding than the intracerebroventricular injection required in other established models of AD-type neuro-degeneration [10,11]. Previous work demonstrated that subcutaneous or oral MSG impaired short term working memory in a T-maze spontaneous alternation paradigm [7].

It has been suggested [12] that species typical-behaviours in rodents can model activities of daily living in humans. The former, including nest-building, digging, burrowing and rearing are greatly impaired by experimental hippocampal lesions [13], and the latter are impaired in AD [14], contributing to a reduced quality of life. In the present study, we investigated whether MSG-treated mice feature tau pathology characteristic of AD in the cortex and hippocampus, and whether this is associated with changes in species-typical behaviour.

## Methods

### Ethic statement

The study protocol was approved by the Ethics Committee of the Faculty of Medicine, University of Alexandria. All applicable international, national, and/or institutional guidelines for the care, use and sacrifice of animals were followed. Additionally, all procedures performed involving animals were in accordance with the ethical standards of Alexandria University, Egypt. Serial no. 0303767–16/11/2017. [IRB NO: 00007555-FWA NO: 00018699].

### Animals

All experimental procedures including termination of the experiment were approved by the Ethics Committee of Alexandria Faculty of Medicine. Cohort sizes were selected using power calculations based on data from pilot studies. Outbred male and female mice derived from the C57BL/6 strain were housed in same sex groups (4 per cage), under a natural light-dark cycle with free access to water and standard lab chow throughout the experiment. Starting at 4 weeks of age, mice in the MSG group (N = 12; 7 male and 5 females) were given a series of subcutaneous injections of MSG (2 g/kg), on alternate days for 10 days; i.e. a total of 5 injections. Controls (N = 12; 6 males and 6 females) received the vehicle, saline. There was no significant difference in the gender distribution between MSG and control groups (P = 0.68 by Fisher’s Exact test). Mice were injected at this relatively older age to avoid the gross motor deficits seen on administration during the first 10 days of life [15]. At age 10–12 weeks, mice were subjected to a battery of behavioural tests following the methods of Deacon et al [13], with slight modifications, to assess different domains of cognitive function and behaviour.

### Behavioural testing

#### Species-typical behaviours

**Nest-building:** Mice were housed overnight in individual cages containing wood chip bedding and one square piece of pressed cotton (5 cm^2^, 0.7 mm in height) with which to make a nest.

The following morning nests were scored according to the following scale [16]:

1. Cotton not noticeably touched (>90% intact).
2. Cotton partially torn up (50–90% remaining intact).
3. Cotton mostly shredded (10–50% intact) but not gathered into a nest.
4. An identifiable but flat nest: <10% cotton intact, but incomplete walls.
5. A (near) perfect nest with walls built up to form a crater: <10% cotton intact.

The scoring was performed independently by 2 researchers blind to group identity, and the average of both assessments was used.

**Burrowing:** The mice were housed in individual cages each containing a burrow consisting of a black plastic tube 20 cm long and 6 cm in diameter, sealed at one end. Burrows were filled with 200 g of food pellets, and left in the cages for 2 h, after which the food pellets remaining in the burrows were weighed, to determine the weight burrowed by the mice [16].

**Marble burying:** Mice spontaneously dig in cage bedding or sand, in a manner that is largely independent of anxiety or novelty [17]. To quantify digging behaviour in the present study, transparent plastic cages (35 x 25 x 25 cm) were filled with sand to a depth of 5 cm, with 12 glass marbles (1.5 cm in diameter) placed evenly in a 3 x 4 array on the surface. Mice were placed individually in the cages for 30 minutes, then the number of marbles buried at least two thirds deep was counted by a researcher blind to group identity.

#### Short-term spatial working memory

T-maze alternation: Rodents have a strong exploratory drive, and this can be used as motivation to perform a short-term spatial working memory test using an enclosed T-maze [13]. If a mouse is allowed to enter one of the cross arms, the second time it is placed in the maze it will generally choose the opposite, non-visited arm; i.e. it will alternate. Each mouse was placed in the start arm, facing away from the choice point (arms junction) and allowed to spontaneously enter a cross arm of its choice (sample arm). The sliding guillotine door of this arm was then closed, and the mouse confined there for 30 s. The mouse was then replaced at the closed end of the start arm, from where it again chose a cross arm. The test was repeated for 10 trials over 5 alternate days, and the % correct alternations calculated.

#### Activity and exploratory behaviour

**Holeboard:** Mice were tested individually on the holeboard, a grey wooden box, 40 × 40 × 27 cm, with the floor raised on 5 cm-long wooden legs. The floor was divided into 16 squares, each 10×10 cm. Four central and 4 peripheral squares contained one 1.5 cm diameter hole each. Mice were placed into a corner and the number of head dips into the holes was counted over 3 min. Head dipping was defined as a hole entry up to the ears [13].

**Open field:** The open field was a black arena, 50×30×18 cm, divided into 10×10 cm squares. The open field is potentially sensitive to both activity and exploration in addition to its use as a measure for anxiety. The individual mouse was placed in a corner square, facing the walls, and observed for 3 min. The following parameters were recorded: latency to leave the first square, total number of squares crossed, latency to the first rear, and the total number of rears [13].

#### Motor coordination

**Static rods:** Mice were tested sequentially on three static rods of 4, 2.5 and 1.0 cm diameter. The rods were horizontally cantilevered by clamping one end 60 cm above a soft surface. Each mouse was placed 2 cm from the free end of the rod, facing away from the supporting bench. The latency to orient 180° to face the bench, and the time taken to transit the rod to the bench, were recorded [13].

### Terminal biochemical and immunohistochemical studies

At age 12 weeks, mice were weighed, then killed by terminal intraperitoneal anaesthesia (ketamine 50 mg/kg + xylazine 5 mg/kg), followed by decapitation. The brains were then excised, and both hemispheres were immediately dissected on ice. The right hemisphere was fixed in 10% formol saline for histological examination. The cortex of the left hemisphere was snap-frozen in liquid nitrogen and stored at -80°C for biochemical assays.

Terminal dissection and weighing of mesenteric (peri-intestinal) and right inguinal fat pad weight were performed to assess visceral and subcutaneous adiposity respectively.

#### Histological examination

After dehydration with a graded ethanol series, frontal cortical and hippocampal tissues were embedded in paraffin wax, and 5-μm-thick sagittal sections were prepared and stained with haematoxylin and eosin (H&E) for histopathological examination.

#### Neuronal phosphorylated tau visualisation by immunohistochemistry

For immunohistochemical detection of abnormally phosphorylated tau, the deparaffinised sections were subjected to heat-induced epitope retrieval in a 700 W microwave oven in 0.01 M citrate buffer (pH 6.0). Hydrogen peroxide (3%) was then applied to block endogenous peroxidase activity. The primary antibody (recombinant rabbit monoclonal anti-Tau (phospho S214) antibody, Cat#ab170892, at a dilution of 1:100 (Abcam, Cambridge, MA, USA) was applied overnight at 4ºC. The UltraVision^TM^ LP Detection System (Cat# TL-015HD) (ThermoFisher Scientific, Waltham, MA, USA) was used for antigen visualization. The immunohistochemical reactions were developed with diaminobenzidine (DAB) chromogen and sections were counterstained with Mayer's haematoxylin.

#### Morphometric studies of immunostained sections

Digital images from immunostained sections were obtained using a camera connected to an Olympus BX41 microscope at a magnification of ×400. Random images were selected and analysed for area percentage of phosphotau S214 immunostaining in the cortex. Measurements were done in NIH Image J (v1.49) (http://rsb.info.nih.gov/ij/) using the Analyze Particles and Colour Deconvolution plugins [18].

#### Tissue neurochemical assays

An enzyme-linked immunosorbent assay (ELISA) was used to measure concentrations of tau protein hyperphosphorylated at threonine 231 (P-tau231P; Elabscience Biotechnology, Wuhan, China) in cortical homogenates, following the manufacturers’ instructions. Colour change was measured spectrophotometrically at wavelength 450 nm. Concentrations were normalized against total protein concentration, measured by modified Lowry method [19].

#### Apoptotic cell counting

H&E-stained sections from the cortex and hippocampus were used to quantify apoptotic cells, which were identified as rounded or oval masses with dark eosinophilic cytoplasm and dense shrunken pyknotic nuclei [20]. The number of apoptotic cells per high-power (x1000) field was counted in 2 representative fields/brain region from each animal in the MSG and control groups, by a pathologist blinded to the experimental groups.

### Statistical analysis

Biochemical, pathological and immunohistochemical data are presented as mean ± SEM and compared by independent samples *t* test. Due to the skewed distribution of several behavioural parameters, non-parametric analysis was used for all behavioural data. Behavioural data are presented as median (25, 75^th^ percentiles), and groups were compared by Mann-Whitney *U* test. To compare the proportions of poor nesters in MSG and control mice, Fisher’s exact test was used. PASW Statistics for Mac (20.0; SPSS Inc., Chicago, IL, USA) and GraphPadPrism (version 6.0f for Mac) were used for analysis and presentation of data. All tests were two-tailed and P <0.05 was considered significant.

## Results

### MSG induced deficits in memory and species-typical behaviours

**Fig 1:**
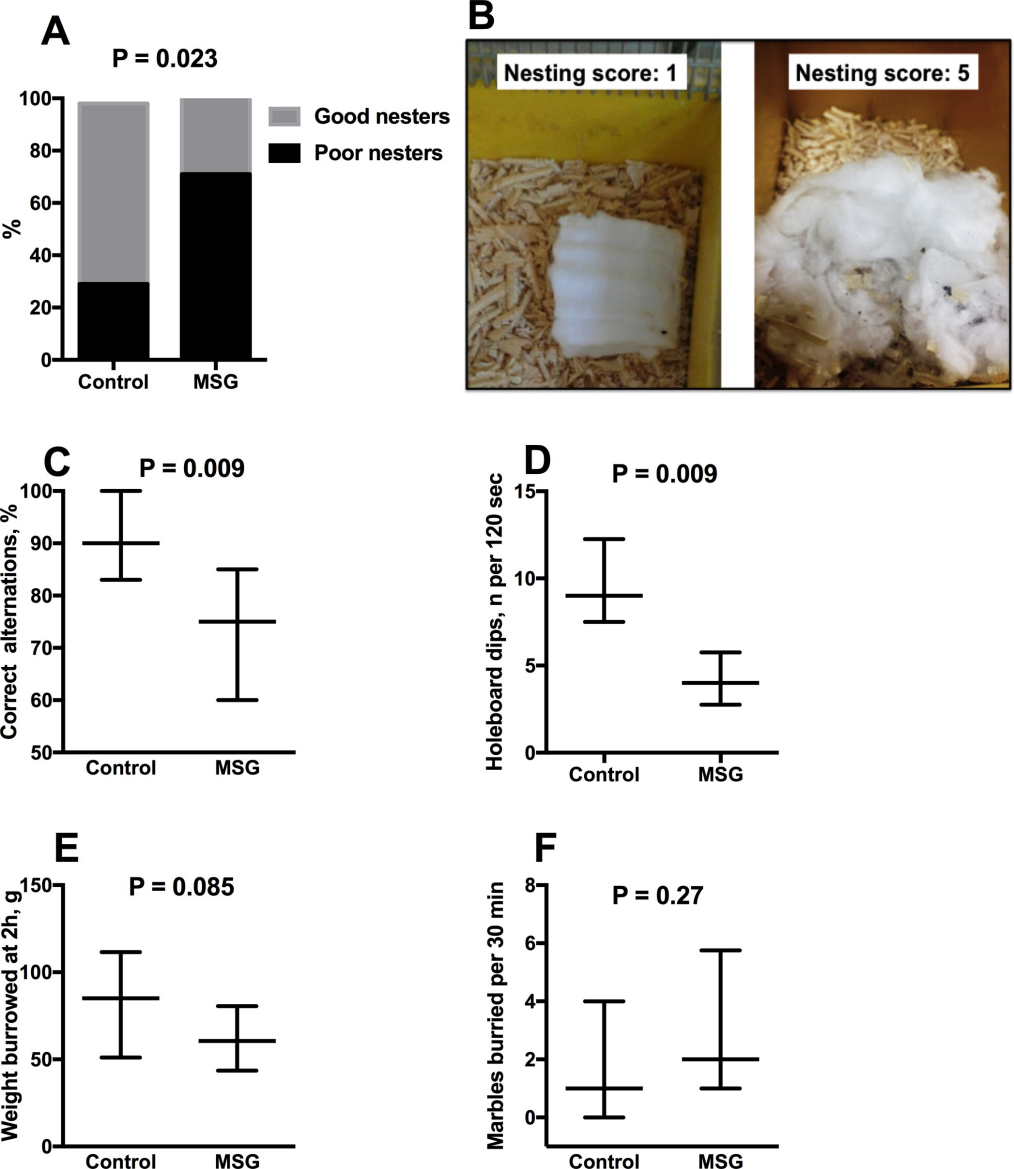
Behavioural test scores in MSG-treated rats and controls. *A;* percentage of poor nesters (see Methods for definition and score details) in monosodium-glutamate (MSG)- treated and control groups at age 10 weeks, compared by Fisher’s exact test. *B;* a typical poor nest (flat, >90% untouched) and a typical good nest (>90% shredded with walls and a central crater) from the MSG and control groups respectively. *C-F*; effect of MSG treatment on spontaneous T-maze alternation (C), and species-typical behavioural test scores *(D-F)* at age 10–12 weeks. Values (C-F) are median (25^th^, 75^th^ percentiles) from N = 12 MSG mice and N = 12 controls. *P* values are from Mann-Whitney *U* test.

Nest building was impaired in MSG-treated mice (Fig 1A). 70% of the MSG-treated mice were “poor nesters”, defined as having a nesting score of 1 or 2 (no nest-like configuration, with 50–100% of the cotton nesting material left intact), compared to 30% of control mice (P = 0.023). A typical “poor nest” (flat, mostly untouched), and good nest (largely shredded with nest walls and a central crater) made by MSG and control mice respectively, are shown in Fig 1B.

Spontaneous T-maze alternation was also lower in MSG-treated mice, indicating impaired short-term working memory (P = 0.009; Fig 1C). MSG treatment also induced a reduction in exploratory behaviour, as indicated by a decrease in the number of head-dips in the holeboard to 44% of control (P = 0.009; Fig 1D). There was a tendency to less burrowing activity in the MSG group (P = 0.085; Fig 1E), but marble burying was not affected by MSG treatment (Fig 1F). Gross motor coordination did not differ in MSG-treated mice from controls, as indicated by their performance on the thick, thin (Table 1) and intermediate (not shown) static rods. There was also no difference in overall activity between MSG-treated and control mice in the dark open field (Table 1).

**Table 1:** Open field and static rods performance in MSG-treated mice and controls^1^.

**Behavioural tests**	**Control**	**MSG**	**P value**
**Open field**			
Latency to 1^st^ crossing, (s)	2 (1, 2)	2 (1, 5)	0.56
Number of crossings	49 (38, 75)	74 (38, 86)	0.37
Latency to 1^st^ rear, (s)	22 (17, 53)	20 (13, 29)	0.15
Number of rears	10 (4, 14)	10 (5, 15)	0.63
**Static rods**	**Control**	**MSG**	**P value**
Thick, time to orient (s)	2 (1, 6)	3 (1, 4)	0.97
Thick, time to base, (s)	19 (12, 26)	9 (8, 14)	0.11
Thin, time to orient, (s)	4 (3, 6)	5 (3, 5)	0.99
Thin, time to base, (s)	21 (10, 40)	11 (7, 14)	0.18

(^1^) Values are median (25^th^, 75^th^ percentile) from N = 12 MSG mice and N = 12 controls. P values are from Mann-Whitney *U* test.

#### MSG treatment did not alter body weight or adiposity

**Fig 2:**
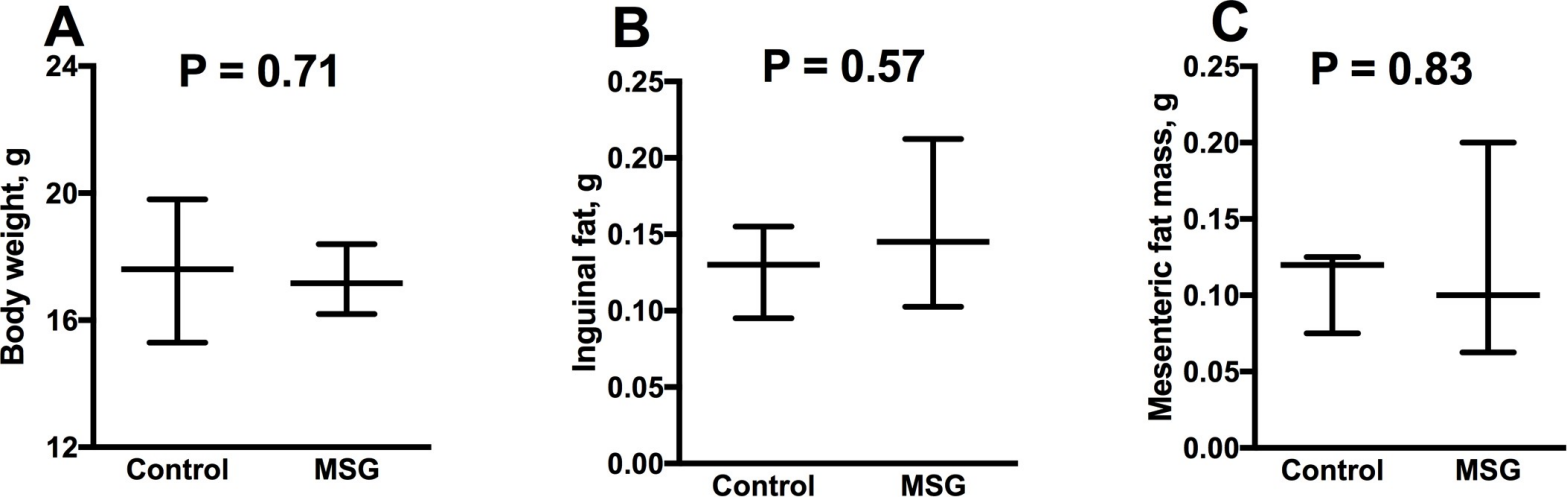
Body weight and composition in MSG-treated mice and controls. Terminal body weight (A) and inguinal (B) and mesenteric fat (C*)* mass in control (N = 12) and MSG (N = 12) mice. Values are median (25^th^, 75^th^ percentiles), and P values are from Mann-Whitney *U* test.

Terminal body weights, and subcutaneous and visceral adiposity, assessed by inguinal and mesenteric (peri-intestinal) fat mass respectively, were not significantly altered by MSG treatment (P ≥ 0.46 for all; Fig 2).

### MSG induced degeneration/apoptosis of cortical neurons and vacuolation of the neuropil

**Fig 3:**
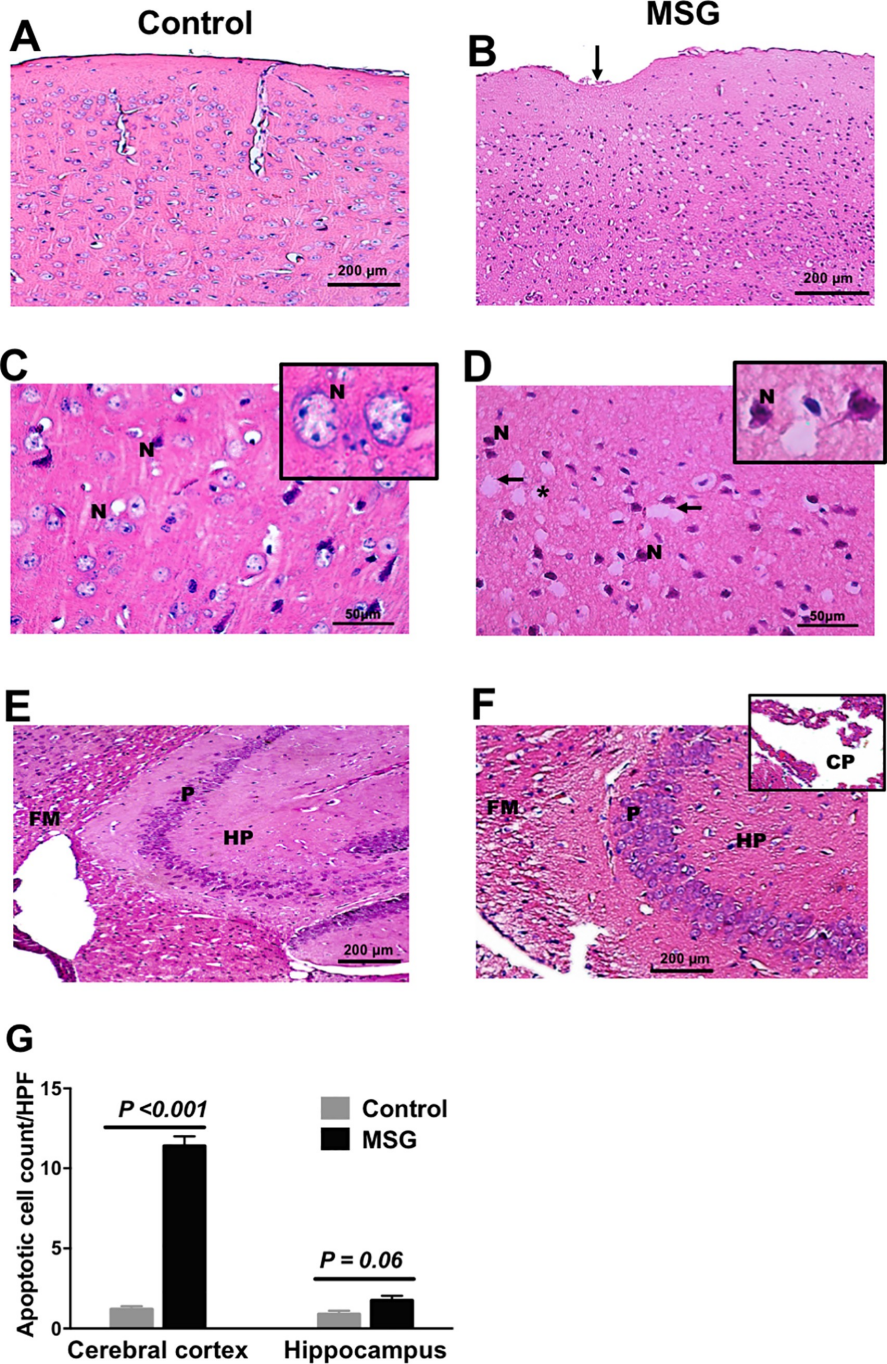
Pathological changes in MSG-treated mouse brains compared to controls. Representative H&E stained photomicrographs of cortical and hippocampal sections from control (left) and MSG-treated mice (right). LEFT: *A*, well-defined layers of cortical neurons from control mice, with a regular superficial molecular layer lying beneath a well-defined cortical surface. *C*, normal-looking cortical neurons with large cell bodies, vesicular nuclei (N) (inset) and a homogeneous compact neuropil. *E*, well- organized pyramidal cell layer (P) of the hippocampus proper (HP) with large vesicular nuclei and triangular cell bodies. Note the uniformly arranged nerve fibres in the fimbria (FM). RIGHT: Equivalent photomicrographs from MSG-treated mice (B, D, F). *B*, depressed, irregular cortical surface (arrow) with disorganized cortical layers. *D*, apoptotic cortical neurons with shrunken cell bodies and dark nuclei (inset). The neuropil appears dysmorphic and extensively vacuolated (arrows) with spongiform neuro-degeneration. *F*, Hippocampal fimbria (FM) exhibits dissolution and breakup of nerve fibre bundles, congestion of the choroid plexus (inset), but apparently normal-looking pyramidal neurons of the hippocampus proper (HP). *G*, Apoptotic cell count in the cortex and hippocampus from MSG-treated and control mice, counted in fields at x1000 magnification.

Fig 3A and 3G shows the pathological changes in H&E-stained sections from the cerebral cortex and hippocampus of MSG-treated mice compared to controls. The cerebral cortex of MSG-treated mice featured evidence of neurodegeneration, with spongiosis of the neuropil, extensive vacuolation, and disorganized cortical laminae (Fig 3D). Cortical neurons showed darkly-stained apoptotic nuclei with areas of neuronal loss, and a nearly 10-fold increase in the number of apoptotic neurons (Fig 3G). Depression of the cortical surface was also noted, suggesting fibrotic changes (Fig 3B). Apoptosis was less pronounced in the hippocampus than in the cortex of MSG-treated mice (Fig 3G), but the hippocampal fimbria showed vacuolation and dissolution of the nerve fibre bundles with congestion of the choroid plexus (Fig 3F). The hippocampal pyramidal neurons were of normal appearance. Cortical and hippocampal sections from control mice showed well-defined layers of cortical neurons with vesicular nuclei and compact neuropils (Fig 3A, 3C and 3E).

### Hyperphosphorylated tau protein was increased in cortical and hippocampal neurons from MSG-treated mice

**Fig 4:**
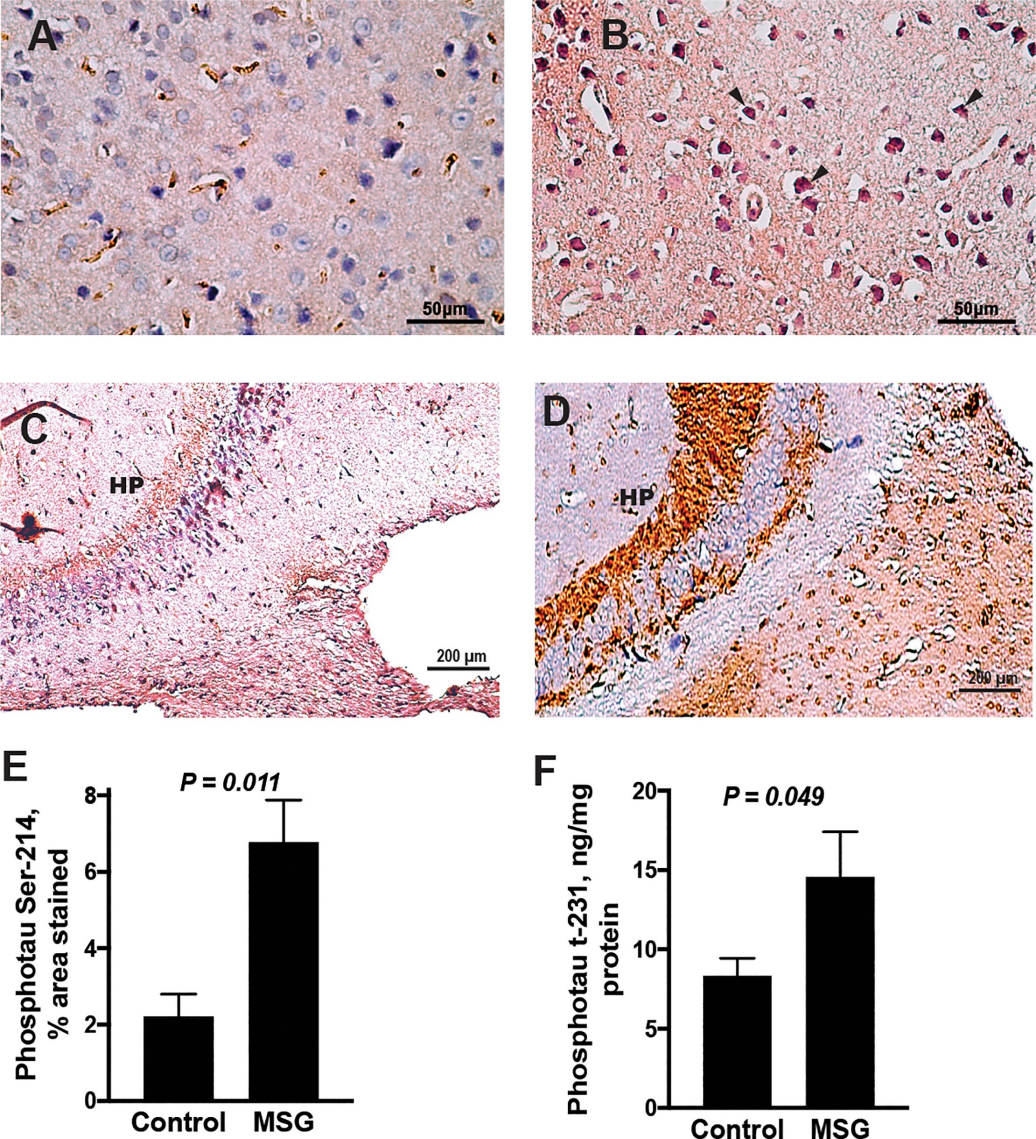
Tau pathology in MSG-treated mouse brains. A-D, representative photomicrographs for Ser-214 phosphorylated tau protein immune-staining, showing cortical (A) and hippocampal (C) neuronal cell bodies of control mice negatively-stained for Ser-214 phosphorylated tau protein, whereas pathologic hyperphosphorylated tau is visible in all layers of the cortex (B) and hippocampal neurons (D) from MSG-treated mice. E: Percent area immunostained with Ser-214 phosphotau antibodies in cerebral cortices from N = 4 MSG-treated and 4 control mice. F: cortical concentrations of phosphorylated tau protein t-231 measured by an ELISA in N = 12 MSG and 12 control mice (F). Data are presented as mean ± SEM and compared by independent samples t test.

Using antibodies specific for tau protein species hyperphosphorylated at Ser-214. MSG-treated mouse cortices showed widespread increase of hyperphosphorylated tau protein in almost all cortical neurons with loss of their laminar pattern (Fig 4B). Intense immunostaining was also revealed in the fimbria in hippocampus proper. The pyramidal neurons of the hippocampus proper, on the other hand showed a non-uniform pattern of staining (Fig 4D). Cortical and hippocampal sections from control mice revealed negatively-stained neuronal cell bodies with basophilic vesicular nuclei arranged in a well-organized compact neuropil (Fig 4A and 4C). Quantitation of the cortical hyperphosphorylated tau load revealed a 3-fold increase in the % area immunostained by the Ser-214 antibody (P = 0.011; Fig 4E). A 43% increase in concentrations of phosphorylated tau protein t-231 measured in cortical homogenates by an ELISA was also observed in the cortex of MSG mice relative to controls (P = 0.049; Fig 4F).

## Discussion

Impairment of short-term memory, and β-amyloid accumulation in the hippocampus, both characteristic of AD, were previously seen in adult rats treated neonatally with MSG [7]. In the present study in mice, we further characterized the MSG neurotoxic model with respect to AD-type tau pathology and effects on species-typical behaviours. MSG-treated mice exhibited diffuse cortical and hippocampal intraneuronal accumulation of hyperphosphorylated tau, with widespread spongiform degeneration and neuronal atrophy. MSG also induced impairments in short-term working memory, nest building and exploratory behaviour. These findings point to MSG as one of the neurotoxins causing AD-like pathology, and raise the question of whether phosphotau accumulation partly underlies some of the previously reported neurological deficits caused by MSG.

MSG is used as a flavor enhancer in human diets, and there is no evidence that its habitual oral use for this purpose has adverse neurological effects [21]. However, it has also been recognized as early as 1969 that parenteral administration of a high dose of MSG to rodents during a narrow window of postnatal development is neurotoxic [6]. Since the initial report [6], MSG neurotoxicity in rodents has been extensively studied using various dosing regimens. Neurotoxic effects of MSG vary widely according to species, dose/route of administration, age at dosing and at subsequent phenotyping. The effects range from learning and locomotor deficits [22–23], to generalized seizures [24], with morphologic and electrophysiological changes in various brain regions [25].

The regimen used in the present study induced cognitive deficits without compromising motor ability. As previously observed in our laboratory in Wistar rats [7], this dose in mice produced significant impairment of working memory, characteristic of AD, in the absence of gross motor abnormalities. There was also impairment of some species-typical behaviours (exploration and nest-building), but not others (digging). The poor nest-building performance is unlikely to have been driven by gender. Both males and female mice were included in the study, and there was no significant difference in gender distribution across the MSG and control groups. To serve the purpose of thermo-regulation, both males and females make nests; and nest scores for males and females tend to be in the same range [16]. With respect to clinical features of AD, the impairment in performing activities of daily living is at least as debilitating as the memory deficit and more strongly correlates with loss of independence [26]. This impairment often precedes deterioration from mild cognitive impairment to AD, and includes compromised performance of tasks such as shopping, self-care, making the bed, using a phone and other modern technology [27]. The corresponding equivalent activities in rodents may include nest building, digging and burrowing [28], which are impaired in the hippocampal cytotoxic lesion model of AD [29].

The selective effect of MSG on hippocampal-dependent behaviours requires interpretation, given that the only species-typical behaviours affected significantly by MSG were nest-building and exploratory behaviour, even though short-term memory was consistently impaired. On one hand, the action of MSG might be characterised as a “mass action” effect. Different behaviours require different amounts of hippocampal functionality, so a task requiring more hippocampal function will be impaired while that requiring less hippocampal input will be spared. Alternatively, a regionally selective theory of hippocampal function might posit that MSG at the present dose selectively impairs only certain hippocampal regions, thus explaining the partial impairment of species typical behaviour. It is well known that the hippocampus is functionally heterogeneous, at least in the rat [30]. Unpublished work (Deacon et al) on mice with selective dorsal, ventral and complete cytotoxic hippocampal lesions suggests that different species-typical behaviours are regionally hippocampal dependent. Clearly, regional hippocampal-behaviour relations warrant further investigation.

Phosphotau accumulation in MSG-treated rodents has been noted in 2 previous studies. Jin et al [31] recently reported hippocampal accumulation of multiple phosphotau isoforms in 3-month old rats given double the MSG dose used in the present study, together with hyperglycemia and AD-like learning and memory deficits with decreased dendritic spine density. Špolcová et al [8] also showed increased tau phosphorylation in MSG-treated mice, but several differences from our study can be noted. In their study, mice were 6 months old, with MSG-induced obesity and prediabetes [8]. Mice in the present study were younger (3 months), and diffuse cortical and hippocampal phosphotau immunoreactivity was observed with no effect on body weight or subcutaneous or visceral adiposity. The lack of an obese phenotype in the present model may be related to the lower dose used and later onset of dosing (2g/kg on 5 alternate days starting at age 4 weeks, compared to 4mg/g/d at postnatal days 2–8 in the Špolcová et al model). We speculate that the later/lower dosing may account for the tau-dependent cortical and hippocampal neurotoxicity observed, while sparing the arcuate nucleus, the damage of which is involved in the hyperleptinemia triggering the MSG-obese phenotype [32]. The tau antibody used in the present study was specific for tau phosphorylated at Ser-214, which stains intraneuronal neurofibrillary tangles, with little affinity for pretangles and neuropil threads [33,34]. The Ser-214 phosphorylation is mediated by cAMP-dependent protein-kinase A and leads to detachment of tau protein from microtubules [35]. Clearly a review of the brain region-specific effects of different MSG-dosing regimens is needed to identify the regimens useful for different disease models.

MSG also increased phosphotau t-231P concentrations in the cerebral cortex of MSG-treated mice, as previously noted by Jin et al in rat hippocampus [31]. Elevation of this tau isoform in human CSF correlates with the cortical load of neurofibrillary tangles and neuritic plaques [36]. T-231P elevation also predicts deterioration from mild cognitive impairment to AD [37], although it was recently shown to accumulate in vascular dementias as well as AD [38]. Tau positive neurons are more susceptible to death [35], which was evidenced in our MSG-treated mice by the marked increase in apoptotic cell count and diffuse spongiform vacuolation in the cortex. Cortical vacuolation is frequently observed in human cases [39], as well as in animal models of AD [40].

The relevance of findings in young mice for an age-related neurodegenerative disease such as AD may be questioned, although MSG is not unique in this respect. The established intra-cerebral streptozotocin model proved useful for studying AD pathology, although the drug is administered at postnatal-day 3, and the AD-type neuro-degeneration is established as early as 21 days of age [10,41]. Another such model is that induced by colchicine in which memory deficits are already present 2 weeks after colchicine injection of young adult rats [11]. In the present model tau hyperphosphorylation, neuropathology and AD-type memory and behavioural deficits we established by 12 weeks of age. These models are all limited by the young age of the animals and by the fact that they may not recapitulate all aspects of AD pathology. Yet they are convenient due to short experimental duration; and are the closest models available for sporadic AD, which comprises approximately 85% of cases; as opposed to familial AD mimicked by transgenic models [33,40].

## Conclusion

MSG administration in early life in mice induced accumulation of hyperphosphorylated tau in the cortex and hippocampus, with diffuse vacuolar changes and neuronal atrophy. These structural abnormalities were associated with impairment of short term-memory and some species-typical behavioural tasks, but not with obesity or locomotor deficits. The findings point to MSG as one of the causes of tau pathology and compromised species-typical behaviour. However, determining whether MSG can be useful in mimicking AD requires further studies of longer duration and fuller behavioural characterization.
